# DNA methylation‐regulated and tumor‐suppressive roles of miR‐487b in colorectal cancer via targeting MYC, SUZ12, and KRAS

**DOI:** 10.1002/cam4.2032

**Published:** 2019-02-21

**Authors:** Xu Chen, Zhi‐feng Lin, Wen‐jin Xi, Wei Wang, Dan Zhang, Fan Yang, Yu‐fang Li, Yi Huo, Tian‐ze Zhang, Yi‐hong Jiang, Wei‐wei Qin, An‐gang Yang, Tao Wang

**Affiliations:** ^1^ State Key Laboratory of Cancer Biology, Department of Immunology Fourth Military Medical University Xi’an, Shaanxi P.R. China; ^2^ Fourth Military Medical University Xi’an, Shaanxi P.R. China; ^3^ Department of Hematology Tangdu Hospital, Fourth Military Medical University Xi’an, Shaanxi P.R. China; ^4^ Department of Medical Genetics and Developmental Biology Fourth Military Medical University Xi’an, Shaanxi P.R. China

**Keywords:** colorectal cancer, DNA methylation, EMT, miR‐487b, tumorigenesis

## Abstract

Human colorectal cancer (CRC), characterized by its high morbidity and lethality, seriously threatens human health and lives. MicroRNA‐487b (miR‐487b) is currently reported to be aberrantly expressed in several tumors, but the detailed functions and underlying mechanisms of miR‐487b in CRC remain unclear. Here, we found that miR‐487b is downregulated in CRC cell lines and is markedly decreased in tumor specimens derived from CRC patients. MiR‐487b inhibits cell proliferation, migration and invasion and promotes the apoptosis of CRC cells in vitro. Statistical analysis of clinical samples indicates that miR‐487b may serve as a biomarker for early CRC diagnosis. Inverse correlations between the expression levels of MYC, SUZ12, and KRAS and that of miR‐487b exist in vitro and in CRC patient tissue specimens. Further experiments demonstrated the regulatory effects of miR‐487b on MYC, SUZ12, and KRAS, and the disruption of these genes partially restores the miR‐487b inhibitor‐induced phenotype. Additionally, miR‐487b promoter region is in a DNA hypermethylated condition and the DNA methyltransferase inhibitor 5‐aza‐2’‐deoxycytidine (5‐Aza) increases the levels of miR‐487b but suppresses the expression of MYC, SUZ12, and KRAS in a time‐ and concentration‐dependent manner in CRC cells. Collectively, miR‐487b is regulated by DNA methylation and it functions as a tumor suppressor in CRC mainly through targeting MYC, SUZ12, and KRAS. Our study provides insight into the regulatory network in CRC cells, offering a new target for treating CRC patients.

## 1 INTRODUCTION

Human colorectal cancer (CRC) is one of the most common digestive malignancies in the world. Accumulated data derived from the World Cancer Trend Analysis showed that CRC is the third most common malignant tumor in males and second most common malignant tumor in females, making it a serious threat to human health and lives.^1^ Characterized by its high recurrence and mortality rate, CRC causes more than 600 000 deaths per year globally.^2^ Metastasis is a pivotal feature that helps CRC cells to survive and escape immune surveillance, exerting negative effects on therapy and the prognosis of CRC patients.^3^ However, the mechanisms underlying CRC metastasis has not yet been fully understood. Therefore, the identification of sensitive biomarkers is of urgent significance for better CRC diagnosis and therapeutics.

MicroRNAs (miRNAs) typically suppress translation or degrade transcripts via direct interactions with the complementary mRNA sequences in 3’‐untranslated regions (3’‐UTRs) during the posttranscriptional phase, and they participate in various biological processes, particularly tumorigenesis.^4^, ^5^ Multiple miRNAs, functioning either as onco‐miRs or tumor suppressors, are implicated in the regulatory networks of CRC development and progression to date.^6^ In addition, aberrant expression of miRNAs in CRC may be attributed to the genetic or epigenetic alterations, such as DNA methylation and histone modification.^7^, ^8^ MiR‐487b, which was first identified as a negative regulator for acute ischemic stroke^9^ and pulmonary fibrosis,^10^ is currently reported to be involved in the modulation of several tumors, such as preventing pulmonary carcinogenesis^11^ and serving as a favorable biomarker for prostate cancer.^12^ Recently, miR‐487b was reported to play a role in regulating CRC tumorigenesis via directly targeting GRM3 and Kirsten rat sarcoma viral oncogene homolog (KRAS).^13^, ^14^ Nevertheless, the function of miR‐487b in CRC and mechanism that accounts for the aberrant expression of miR‐487b in tumors are not fully elucidated. Further investigations are needed to fill in these blanks.

Epithelial‐mesenchymal transition (EMT), a canonical process of morphology alteration in diverse tumors, is closely associated with the malignant phenotypes of cancer cells and significantly facilitates tumor transformation and development.^15^ Disruption of epithelial marker cadherin 1, type 1, E‐cadherin (epithelial) (CDH1) and enhancement of mesenchymal marker Vimentin are critical molecular changes during EMT.^16^ Various signaling pathways, including transforming growth factor beta (TGF‐β), tyrosine kinase receptor (RTK), Notch and wingless integrated (Wnt) pathways,^17^ as well as miRNAs—for example, miR‐122,^18^ miR‐145,^19^ and miR‐154^20^—directly or indirectly regulate the expression of CDH1, Vimentin or the entire process of EMT.^21^ Note, miR‐154 gene family members miR‐300^22^ and miR‐369^23^ have been verified to participate in the EMT in several cancers. Thus, we hypothesize that, as a member of the miR‐154 gene family, miR‐487b also likely plays a similar role in the regulation of EMT.

In this study, we demonstrated that endogenous miR‐487b inhibition is an advantageous condition for CRC cells to transfer from the epithelial phenotype to mesenchymal phenotype and acquire the capability to proliferate, migrate, and invade uncontrollably. Mechanistically, miR‐487b reduced the expression of v‐myc avian myelocytomatosis viral oncogene homolog (MYC), suppressor of zeste 12 protein homolog (SUZ12) and KRAS, and blocked the development of EMT via increasing CDH1 and decreasing Vimentin in vitro and in vivo. Additionally, our studies indicated that DNA methylation might be the reason for the aberrantly low expression of miR‐487b in CRC. In summary, we identified miR‐487b as a CRC suppressor and provide a novel target for CRC diagnosis and therapy.

## 2 MATERIALS AND METHODS

### 2.1 CRC patient specimens

Forty‐one CRC patients with matched adjacent normal mucosae, primary tumors, and metastatic tissues derived from the lymph nodes or hepatocellular carcinomas were collected from the Gastroenterology Department of Xijing Hospital affiliated with the Fourth Military Medical University (FMMU). All the patients included in this study had definite pathological diagnoses and underwent surgery on primary and metastatic tumors from 2008 to 2015. Our research was approved by the Medical Ethics Committee of FMMU, and written informed consent was obtained from all patients ahead of the experiments. Information on these CRC patients is listed in the Supporting information section (Table S1).

### 2.2 Cell cultivation and transient transfection

The normal colorectal cell line, HIEC and CRC cell lines, HCT116, HT29, SW480, and SW620 were all purchased from the Cell Bank of Chinese Academy of Science (SIBS, Shanghai, China). Authentications by STR profiling of all the above cell lines were performed in the Center for DNA Typing of the Fourth Military Medical University (FMMU). For cell cultivation, HIEC cells were cultured in Roswell Park Memorial Institute‐1640 (RPMI‐1640) medium (Gibco, Los Angeles). HCT116 and HT29 cells were cultured in McCoy's 5A medium (Gibco). SW480 and SW620 cells were cultured in Leibovitz's L‐15 medium (Gibco). All of the mediums were supplemented with 10% fetal bovine serum (FBS) (Gibco) and 1% penicillin‐streptomycin. The cells were maintained in a constant 5% CO_2_, 37°C incubator (Thermo, MA). Transient transfection was performed according to manufacturer's instructions by using the Lipofectamine 2000 Transfection Reagent (Invitrogen, Carlsbad). Oligonucleotides were transfected at a final concentration of 2 μg/mL for 48 h. Cells were treated with 5‐aza‐2’‐deoxycytidine (5‐Aza) (Sigma‐Aldrich, Santa Clara) for the in vitro assays. Information on oligonucleotides and reagents is listed in the Supporting information section (Table S3 and S5), respectively.

### 2.3 RNA isolation, reverse transcription, and quantitative real‐time polymerase chain reaction (qRT‐PCR)

Total RNA of cells was extracted by TRIzol (Invitrogen) and sequentially purified via the chloroform, isopropanol, and 70% ethanol. Reverse transcription for miRNA and mRNA relied on a SYBR^®^ PrimeScript™ miRNA RT‐PCR Kit and PrimeScript™ RT Master Mix (TaKaRa, Shiga, Japan), respectively. Quantitative real‐time polymerase chain reaction (qRT‐PCR) was applied on Bio‐Rad CFX96 system (Bio‐Rad, Hercules) with FastStart Essential DNA Green Master (Roche, Indianapolis). The U6 and β‐actin were used to normalize the miRNA and mRNA samples, respectively. Relative quantification of target primers was calculated by the 2^−ΔΔCT^ method. All experiments were repeated in triplicate. Primers used for qRT‐PCR analysis are listed in the Supporting information section (Table S2).

### 2.4 Protein isolation and Western blot analysis

Total protein of cell lysates was isolated by the RIPA lysis buffer (Genshare, Xi'an, China) and quantified via a bicinchoninic assay (BCA). Twenty‐five micrograms of protein samples was separated by the 10% SDS‐PAGE gel and then transferred onto the polyvinylidene fluoride (PVDF) membranes (Millipore, Billerica). After 1 hour block through 5% BSA at room temperature, the PVDF membranes were incubated with the appropriate primary antibodies at 4°C overnight. The PVDF membranes were then washed with TBST (Tris Buffered Saline with 0.05% Tween‐20) multiple times and incubated with the secondary antibodies at room temperature for 1 hour. FluorChem FC2 system (Alpha Innotech, San Leandro) was used to measure the protein signals with different exposure time ranging from 1‐60 s. Antibodies used for Western blot analysis are listed in the Supporting information section (Table S4).

### 2.5 Cell proliferation assays

Proliferative ability of CRC cells was analyzed by MTT and colony formation assays. Briefly, for the MTT assay, 2 × 10^3^ cells were seeded into the 96‐well plates in 200 μL serum medium. Twenty microliters of MTT was added to each well and incubated with the cells at 37°C, 5% CO_2_ for 4 hours. Supernatant was removed by a vacuum extractor and 150 μL dimethylsulfoxide (DMSO) was added to dissolve the cell lysates. Single absorbance at 490 nm was examined by a multiwell plate reader (Bio‐Rad). Each experiment was performed in six repetitions. For the colony formation assay, 2 × 10^3^ cells were plated into the 6 cm‐dishes with 10 mL serum medium at 37°C, 5% CO_2_ for 10‐14 days. Then, cells were washed with phosphate buffer saline (PBS) multiple times and fixed by 5 mL methyl alcohol for 20 minutes. Colonies were stained with Giemsa and visually counted behind a transparent mash paper. Each experiment was repeated in triplicate.

### 2.6 Cell cycle and apoptosis analysis

Flow cytometry was used to determine the cell cycle distribution and cell apoptosis. Briefly, for cell cycle analysis, 1 × 10^6^ cells were washed three times with PBS and were fixed in 70% ethanol at 4°C overnight. Fixed cells were then stained with propidium iodide (PI) for 2 hours at 4°C in the dark. The distribution and percentage of cells in different cycle phases were analyzed by CellQuest software. For cell apoptosis analysis, 1 × 10^6^ cells were harvested and washed three times with PBS. According to the manufacturer's instructions, the cells were then stained with an Annexin V‐FITC plus PI mixture. Cell apoptosis was detected at 488 nm and was analyzed using CellQuest software.

### 2.7 Cell migration and invasion analysis

Wound‐healing and Transwell assays were performed to examine the migration and invasion of CRC cells in vitro. Briefly, for the wound‐healing assay, a 100‐μL pipette tip was applied to scratch the monolayer of cells that were cultivated to 90% confluence vertically. The created wound was observed and photographed under a microscope at the indicated times (0, 24, 48 and 72 hours). For the Transwell assay, 200 μL serum‐free medium containing resuspended 1 × 10^4^ cells were plated into the upper chamber membrane (24‐well insert; pore size, 8 μm; Millipore) with 500 μL serum medium in the lower chamber. After 24‐36 hours of incubation at 37°C, the cells were fixed with 4% paraformaldehyde, stained with 0.1% crystal violet, and counted under a light microscope. Each experiment was performed at least in triplicate.

### 2.8 Pyrosequencing analysis

Genomic DNA from the human tissues was extracted using the Blood and Tissue DNA Kit (Qiagen, Hilden, Germany) and was subjected to bisulfite treatment using the EpiTect Bisulfite Kit (Qiagen) according to the manufacturer's instructions. Next, the genomic DNA samples were PCR amplified and the site‐specific methylation levels were quantified via pyrosequencing analysis. Briefly, the sequencing samples were first prepared using the Vacuum Prep workstation (Biotage, Uppsala, Sweden) and then were transferred to a plate harboring 0.4 μmol/L of sequencing primers in 40 μL of annealing buffer, followed by heating at 80°C for 2 minutes. Pyrosequencing analysis was performed using the PyroMark Gold Q96 Reagent and PyroMark ID System (Qiagen), and the results were analyzed using Pyro Q‐CpG™ software v. 1.0.9 (Sangon Biotech, Shanghai, China). The primer sequences of the pyrosequencing analysis are listed as follows:

Primer 1: F 5′‐GTTAAAAGTATGTAYGATGTGTGTGG‐3′

R 5′‐ATAACAACAAAAACCACAAAACC‐3′

Primer 2: F 5′‐TTGTTGGGGTTGAAYGAGTTAAG‐3′

R 5′‐AACACACAAAAATCCTAACTACCAC‐3′

### 2.9 Statistical analysis

Statistical analysis was performed using SPSS 17.0 software. The data are represented as the means ± standard division (SD) of at least three independent experiments. Student's *t *test was used to evaluate the statistical significance between two independent samples or groups. Analysis of more than two groups was performed by one‐way analysis of variance. Spearman's rank correlation test was applied to evaluate the correlation significance between miRNA and mRNA. Receiver operating characteristic (ROC) curves were used to assess diagnostic accuracy through area under the curve (AUC) analysis. A difference was considered statistically significant when *P < 0.05, **P < 0.01 and ***P < 0.001.

## 3 RESULTS

### 3.1 MiR‐487b is aberrantly expressed in CRC cell lines and prevents the abnormal proliferation of CRC cells in vitro

**Figure 1:**
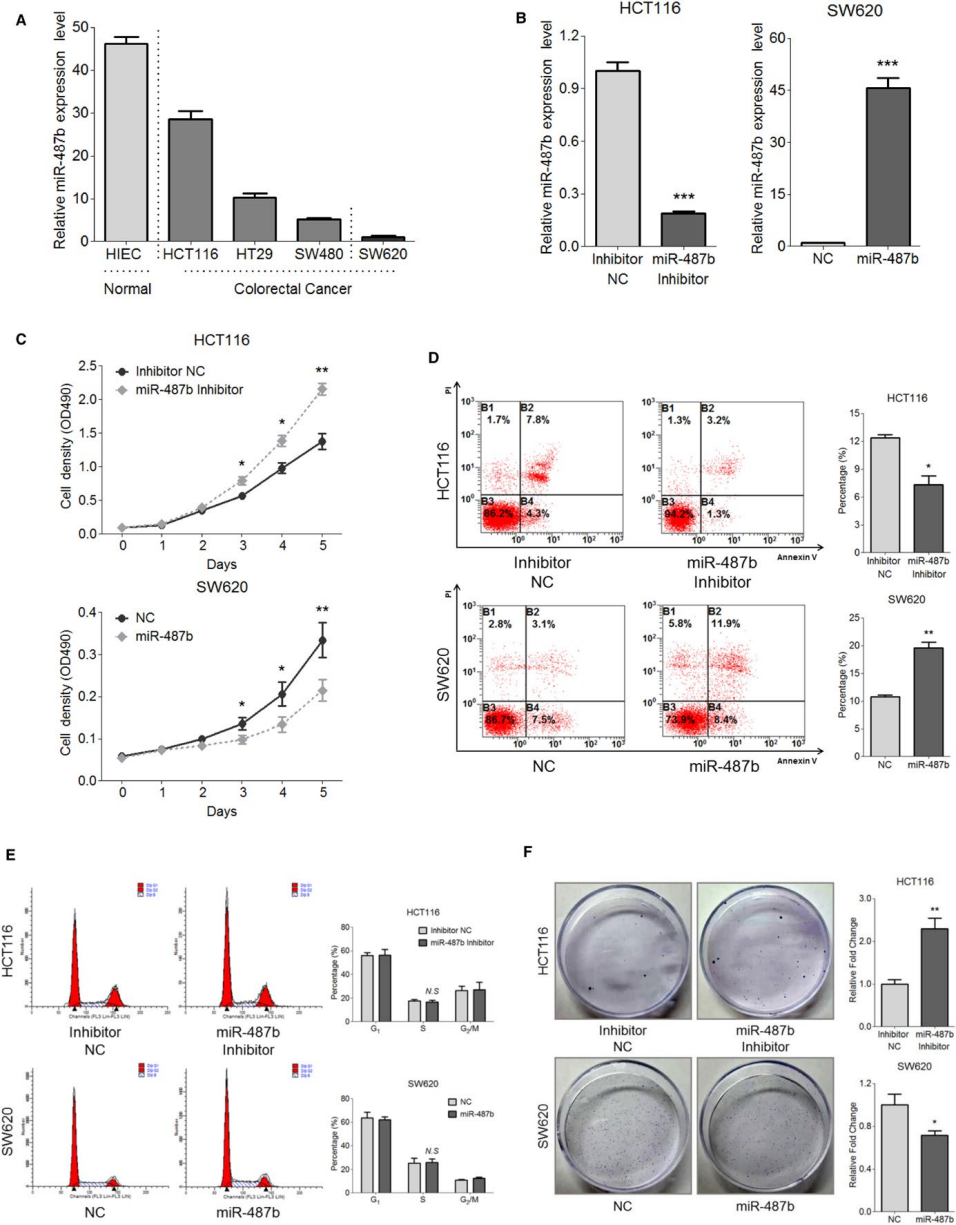
MiR‐487b is downregulated in colorectal cancer (CRC) cells and inhibits CRC cell proliferation. A, The expression level of endogenous miR‐487b was measured in five colorectal cell lines. Vertical dotted lines are used to differentiate normal epithelial, tumor epithelial, and mesenchymal cells from each other. B, qRT‐PCR analysis for the efficiency of miR‐487b knockdown in HCT116 (*left*) cells and overexpression in SW620 (*right*) cells with the indicated oligonucleotide transfection. C, The proliferative ability of HCT116 miR‐487b inhibitor/NC (*upper*) cells and SW620 miR‐487b mimic/NC (*lower*) cells was measured via the MTT assay. D and E, Flow cytometry analysis was used to determine the cell apoptosis (D) and cell cycle (E) of HCT116 miR‐487b inhibitor/NC cells (*upper*) and SW620 miR‐487b mimic/NC cells (*lower*). F, The HCT116 and SW620 cells with the indicated transfection were subjected to the colony formation assay. The data are represented as the means ± SD of no less than three independent experiments. NS, no significance, *P < 0.05, **P < 0.01 and ***P < 0.001

To determine the endogenous miR‐487b expression and select experimental subjects, we first performed qRT‐PCR analysis on five different colorectal cell lines. As shown in Figure 1A, HIEC, a normal intestinal epithelial cell line, harbored a higher miR‐487b level than that of the other four CRC cell lines. Additionally, miR‐487b was predominantly expressed in three epithelial CRC cells (HCT116, HT29, and SW480) but was barely detectable in mesenchymal CRC cells (SW620). Moreover, among the three CRC cell lines with elevated miR‐487b expression, SW480 belongs to the Dukes grade B group that lacks invasiveness, while the lowest miR‐487b‐expressing cell line, SW620, is featured by high metastasis potential and identified as Dukes grade C.^24^ These results support the hypothesis that miR‐487b is markedly inhibited in CRC cell lines and serves as a metastasis suppressor in vitro.

Based on the detection results in CRC cell lines, we next performed a series of gain‐ and loss‐of‐function assays in HCT116 and SW620 cells to explore what role, if any, miR‐487b plays in CRC tumorigenesis. Because miR‐487b was relatively higher in HCT116 cells than in SW620 cells (Figure 1A), we knocked down miR‐487b in HCT116 cells via its inhibitor and enhanced miR‐487b in SW620 cells by its mimic compared with each NC group (Figure 1B). An MTT assay was applied to determine the effects of miR‐487b on CRC cell proliferation. As shown in Figure 1C, the proliferation ability of HCT116 cells was elevated with the downregulation of miR‐487b, but the amplified proportion of SW620 cells was decreased via miR‐487b overexpression. In addition, cell apoptosis analysis through flow cytometry exhibited that the miR‐487b inhibitor‐treated HCT116 cells resulted in a remarkable decline in cell apoptosis, whereas the opposite result was observed in miR‐487b mimic‐treated SW620 cells (Figure 1D). However, significant changes in the cell cycle did not appear in the HCT116 miR‐487b inhibitor/NC or SW620 miR‐487b mimic/NC cells (Figure 1E), indicating that the miR‐487b‐induced CRC cell proliferation limitation mainly relied on its stimulatory effect on apoptosis rather than on cell cycle arrest. Furthermore, in agreement with the MTT assay, colony amounts of the HCT116 cells were notably boosted with the transient transfection of the miR‐487b inhibitor compared with that in the NC group, and vice versa in SW620 miR‐487b mimic/NC cells (Figure 1F). Taken together, these findings demonstrate that miR‐487b can prevent the proliferation of CRC cells in vitro, as suggested by the data acquired from HCT116 and SW620 cells.

### 3.2 MiR‐487b is downregulated in metastatic CRC specimens and suppresses the migration and invasion of CRC cells

**Figure 2:**
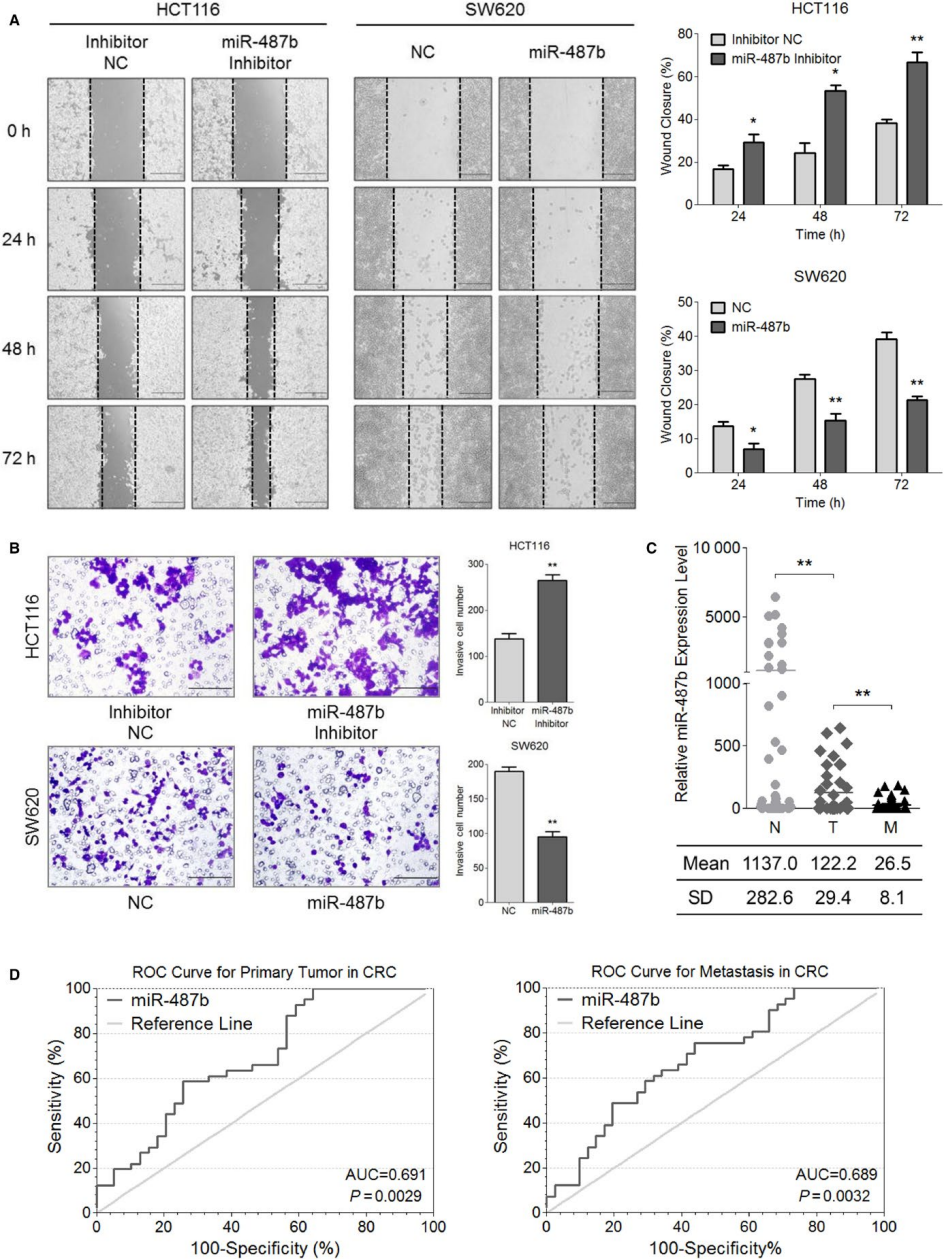
MiR‐487b serves as an antimetastatic regulator and a potential diagnostic biomarker in colorectal cancer (CRC). A, The wound‐healing assay was performed in HCT116 miR‐487b inhibitor/NC cells and SW620 miR‐487b mimic/NC cells to assess cell migration for 0, 24, 48, and 72 h (*left*). Statistical analysis (*right*) for HCT116 (*upper*) and SW620 (*lower*) cells is shown. The *Scale bars* represent 200 μm. B, The migratory and invasive ability of HCT116 and SW620 cells with indicated transfection was evaluated via Transwell experiments. The *Scale bars* represent 100 μm. C, MiR‐487b was differentially expressed in normal (N, 1137.0 ± 282.6), tumor (T, 122.2 ± 29.4), and metastatic (M, 26.5 ± 8.1) tissues as determined by qRT‐PCR analysis. D, Receiver operating characteristic (ROC) curve analysis for the accuracy of miR‐487b in the diagnosis of primary tumor (l*eft*) and metastasis (r*ight*) in CRC. The data are shown as area under the curves (AUCs). The data are presented as the means ± SD of three independent experiments. *P < 0.05 and **P < 0.01

In accordance with the endogenous miR‐487b level in CRC cell lines (Figure 1A), we speculated that miR‐487b might play an antimetastasis role because it was restrained in mesenchymal SW620 cells and elevated in epithelial cells (HIEC, HCT116, HT29, and SW480). To investigate this assumption, we conducted wound‐healing and Transwell assays in HCT116 and SW620 cells. Knockdown of miR‐487b significantly promoted healing (Figure 2A) and invasion through the Transwell membranes of the chambers (Figure 2B) in HCT116 cells. In contrast with the observations of HCT116 cells, overexpression of miR‐487b markedly prevented SW620 cells from migrating into a monolayer of the wounded cells (Figure 2A) and abolished the invasive cell amounts onto the other side of Transwell membranes (Figure 2B). These results support miR‐487b participation in repressing the metastasis and invasiveness of CRC cells in vitro.

Although the anti‐CRC role of miR‐487b was validated through a series of in vitro experiments according to our present data, the in vivo function of miR‐487b remained unclear. Therefore, we measured the expression of miR‐487b in 41 matched adjacent normal mucosae (N), primary CRC tissues (T) and metastasis tissues derived from the lymph nodes or hepatic tumors (M) from Xijing Hospital using qRT‐PCR. The results showed that miR‐487b was significantly upregulated in group N (1137.0 ± 282.6) compared with group T (122.2 ± 29.4) and M (26.5 ± 8.1) (Figure 2C), indicating the antitumor and antimetastasis functions of miR‐487b in CRC in vivo. Next, receiver operating characteristic (ROC) curve analysis was performed depending on the miR‐487b level in CRC patient specimens to further evaluate the association between miR‐487b and the primary tumor or metastasis. Intriguingly, we found that miR‐487b might be an effective diagnosis biomarker to differentiate between the occurrence of the primary tumor, with an area under the curve (AUC) of 0.691 (*P = *0.0029), and metastasis, with an AUC of 0.689 (*P = *0.0032) (Figure 2D). These data clarify an anti‐CRC role, especially an antimetastatic role, of miR‐487b in vivo, and its potential clinical value for CRC.

### 3.3 MiR‐487b positively correlates with CDH1 expression and suppresses the process of EMT

**Figure 3:**
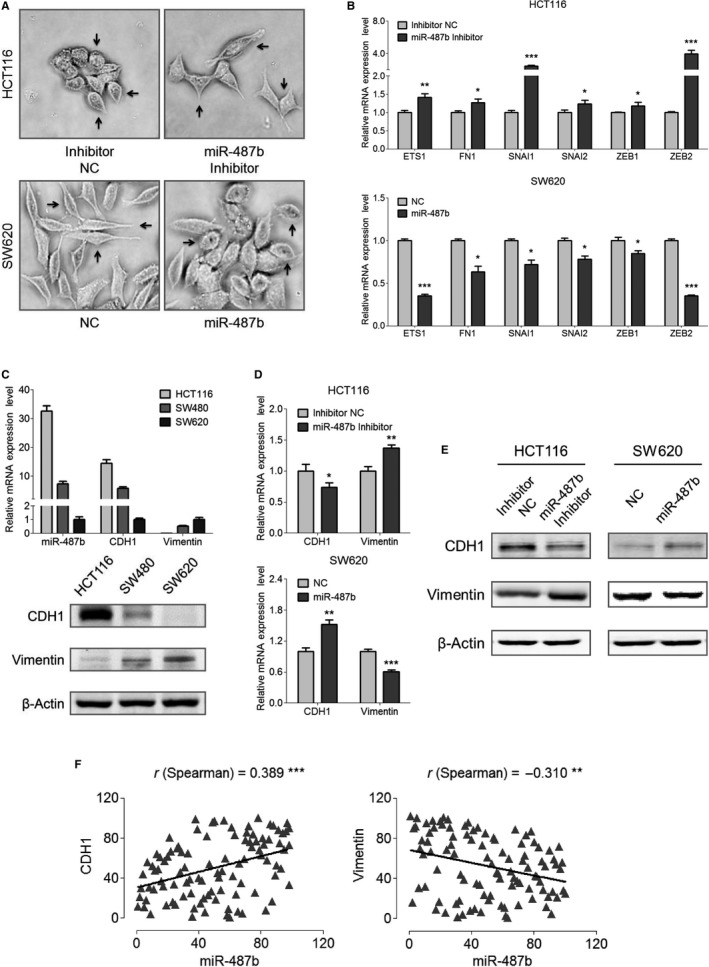
MiR‐487b blocks the development of epithelial‐mesenchymal transition (EMT). A, The altered morphology of HCT116 miR‐487b inhibitor/NC cells and SW620 miR‐487b mimic/NC cells was observed under a light microscope. Representative alterations of morphology are marked by *black arrows*. B, The mRNA levels of six EMT hallmarks in the indicated miR‐487b oligonucleotide‐treated HCT116 and SW620 cells. C, Endogenous expression of miR‐487b, CDH1, and Vimentin in HCT116, SW480, and SW620 cells was tested by qRT‐PCR (*upper*) and Western blot analysis (*lower*). D and E, The expression of CDH1 and Vimentin in HCT116 miR‐487b inhibitor/NC cells and SW620 miR‐487b mimic/NC cells was measured via qRT‐PCR (D) and Western blot analysis (E). F, Spearman's rank correlation test was applied to evaluate the probable relationship between miR‐487b and CDH1 as well as between miR‐487b and Vimentin. The data are shown as the means ± SD of three independent experiments. *P < 0.05, **P < 0.01, and ***P < 0.001

To further investigate the modulation that miR‐487b inhibits CRC metastasis, we first discovered the morphological alterations of HCT116 and SW620 cells by the indicated oligonucleotide transfection under a microscope. As shown in Figure 3A, knockdown of miR‐487b in HCT116 cells caused a loose cell‐cell adhesion and fibroblast‐like change, whereas SW620 cells with miR‐487b overexpression appeared to form tight junctions in contact with each other and a reverse mesenchymal phenotype, suggesting the potential anti‐EMT function of miR‐487b. Next, alterations in EMT hallmarks were further assessed following the intracellular up‐ or downregulation of miR‐487b in SW620 and HCT116 cells. The EMT hallmarks—for example, the ETS proto‐oncogene 1 (ETS1), fibronectin 1 (FN1), snail family transcriptional repressor 1, 2 (SNAI1 and SNAI2), and zinc finger E‐box binding homeobox 1, 2 (ZEB1 and ZEB2)—exhibited different degrees of elevation when miR‐487b was decreased in HCT116 cells and generally descended concomitantly with the increase in miR‐487b in SW620 cells (Figure 3B), providing evidence for the identification of miR‐487b as an EMT suppressor in CRC cells.

The epithelial marker CDH1 and the mesenchymal marker Vimentin are pivotal hallmarks of the EMT process. We first compared the endogenous expression levels of these two hallmarks in HCT116, SW480, and SW620 cells. Our experiments showed that miR‐487b had the highest expression in HCT116 and the lowest expression in SW620 along with moderate expression in SW480, a finding that was consistent with the expression trend of CDH1 and opposite trend of Vimentin (Figure 3C). Next, we detected the expression levels of CDH1 and Vimentin in HCT116 miR‐487b inhibitor/NC cells and SW620 miR‐487b mimic/NC cells at both mRNA (Figure 3D) and protein (Figure 3E) levels. The results showed that reducing miR‐487b in HCT116 cells induced the elevation of Vimentin and decline of CDH1, whereas enhancement of miR‐487b mediated an opposite effect on CDH1 or Vimentin in SW620 cells. Finally, we examined the levels of CDH1 and Vimentin in the 41 matched CRC patient specimens to clarify the potential relationship between miR‐487b and these two EMT hallmarks. Spearman's rank correlation test analysis showed that miR‐487b expression was positively related to CDH1 (r = 0.389, ***P < 0.001) but negatively associated with Vimentin (r = −0.310, **P < 0.01) (Figure 3F). Taken together, these results suggest a positive correlation between miR‐487b and CDH1, thus further highlighting the anti‐EMT role of miR‐487b.

### 3.4 MiR‐487b is an endogenous inhibitor of MYC, SUZ12, and KRAS in CRC cells and patient specimens

**Figure 4:**
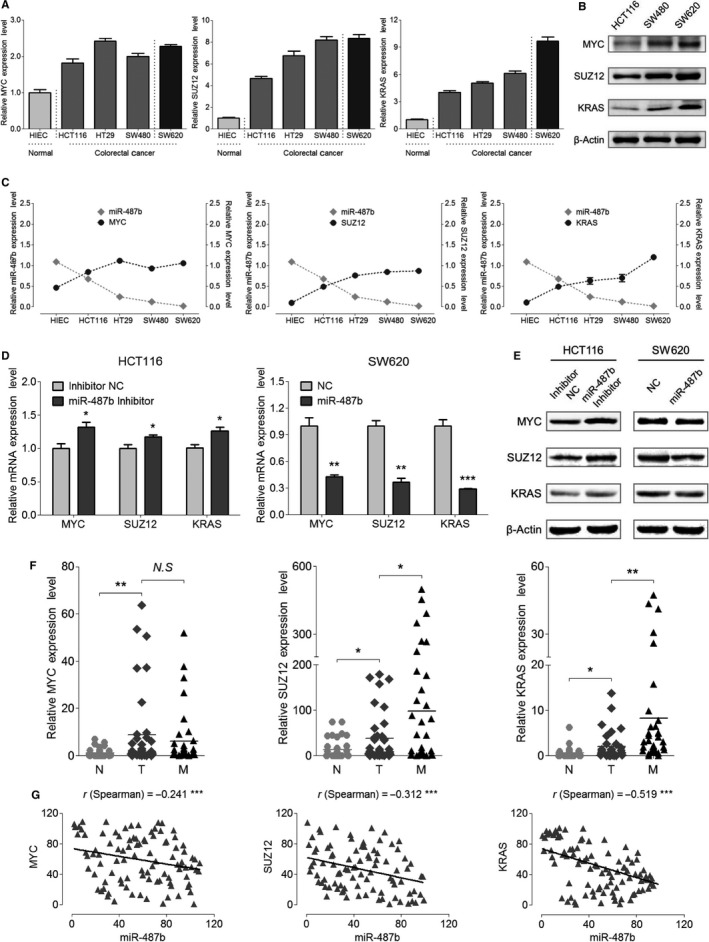
MYC, SUZ12, and KRAS are the targets of miR‐487b in colorectal cancer (CRC) cells and patient specimens. A, Expression levels of MYC, SUZ12, and KRAS mRNA in HIEC, HCT116, HT29, SW480, and SW620 cells were detected by qRT‐PCR. B, Endogenous protein levels of MYC, SUZ12, and KRAS in HCT116, SW480, and SW620 cells were tested via Western blot analysis. C, Comparisons between the expression curves of miR‐487b and MYC/SUZ12/KRAS in the five colorectal cell lines. (D and E) The expression of MYC, SUZ12, and KRAS was examined following the transfection of the miR‐487b inhibitor in HCT116 cells and miR‐487b mimic in SW620 cells compared with that in each NC group at the mRNA (D) and protein (E) levels. F, Expression levels of MYC, SUZ12, and KRAS in the 41 matched CRC patient specimens. G, The correlation between miR‐487b and MYC/SUZ12/KRAS was evaluated by the Spearman's rank correlation test. Error bars represent the SD of three independent experiments. NS, no significance, *P < 0.05, **P < 0.01, and ***P < 0.001

A previous study has reported that MYC, SUZ12, and KRAS are the biotargets of miR‐487b in cigarette smoke‐induced lung cancer,^11^ leading us to speculate that miR‐487b could also suppress CRC tumorigenesis by inhibiting these three genes. The expression patterns of endogenous MYC, SUZ12, and KRAS in colorectal cells revealed an analogous trend via qRT‐PCR analysis. The normal cells, HIEC, possessed the lowest levels of MYC, SUZ12, and KRAS, while the mRNAs of these genes were dramatically elevated in SW620 cells. Among the three cell lines with moderate miR‐487b levels, MYC, SUZ12, or KRAS levels were increased in HCT116, HT29, and SW480, in that order (Figure 4A). MYC, SUZ12, and KRAS levels were also markedly upregulated in SW620 cells compared with those in both HCT116 cells and SW480 cells at the protein level (Figure 4B). Therefore, a strong inverse correlation between miR‐487b and MYC, SUZ12, or KRAS was observed by overlapping the miR‐487b broken line with those of the three targets (Figures 1A and 4C). Additionally, we knocked down or overexpressed miR‐487b in HCT116 and SW620 cells to discuss the exogenous impact on the expression levels of MYC, SUZ12, and KRAS. As shown in Figure 4D, repressing miR‐487b by a pool of inhibitors led to promotion in MYC, SUZ12, and KRAS levels in HCT116 cells, while enhancing miR‐487b via its mimic in SW620 cells significantly restrained the expression of these three genes at the mRNA level. Western blot analysis displayed a similar result in CRC cells with the indicated transfections (Figure 4E). In summary, these data strongly exhibit an inverse relationship between miR‐487b and MYC/SUZ12/KRAS in CRC cells in vitro.

Additional experiments were performed to investigate the negative correlation between miR‐487b and its three targets in vivo. We detected the distribution of MYC, SUZ12, and KRAS in patient samples from the 41 paired normal (N), tumorous (T), and metastatic (M) tissues, respectively. Consistent with the colorectal cell lines (Figure 4A), MYC, SUZ12, and KRAS were all upregulated in the primary tumors compared with those in normal tissues, indicating the intrinsic oncogenes of these biotargets in CRC. Additionally, the expression levels of both SUZ12 and KRAS were pronouncedly higher in metastatic tissues than in primary tumor tissues, suggesting the functions of SUZ12 and KRAS were also closely metastasis related (Figure 4F). Next, the association between miR‐487b and these three genes was further analyzed by Spearman's rank correlation test analysis. The correlation coefficients of miR‐487b and its targets, MYC (r = −0.241, ***P < 0.001), SUZ12 (r = −0.312, ***P < 0.001), and KRAS (r = −0.519, ***P < 0.001), were revealed when plotted with each other (Figure 4G). In conclusion, these results demonstrate MYC, SUZ12, and KRAS as intrinsic biological targets of miR‐487b within the inhibition of CRC progression.

### 3.5 MYC, SUZ12, and KRAS participate in miR‐487b‐induced CRC suppression

**Figure 5:**
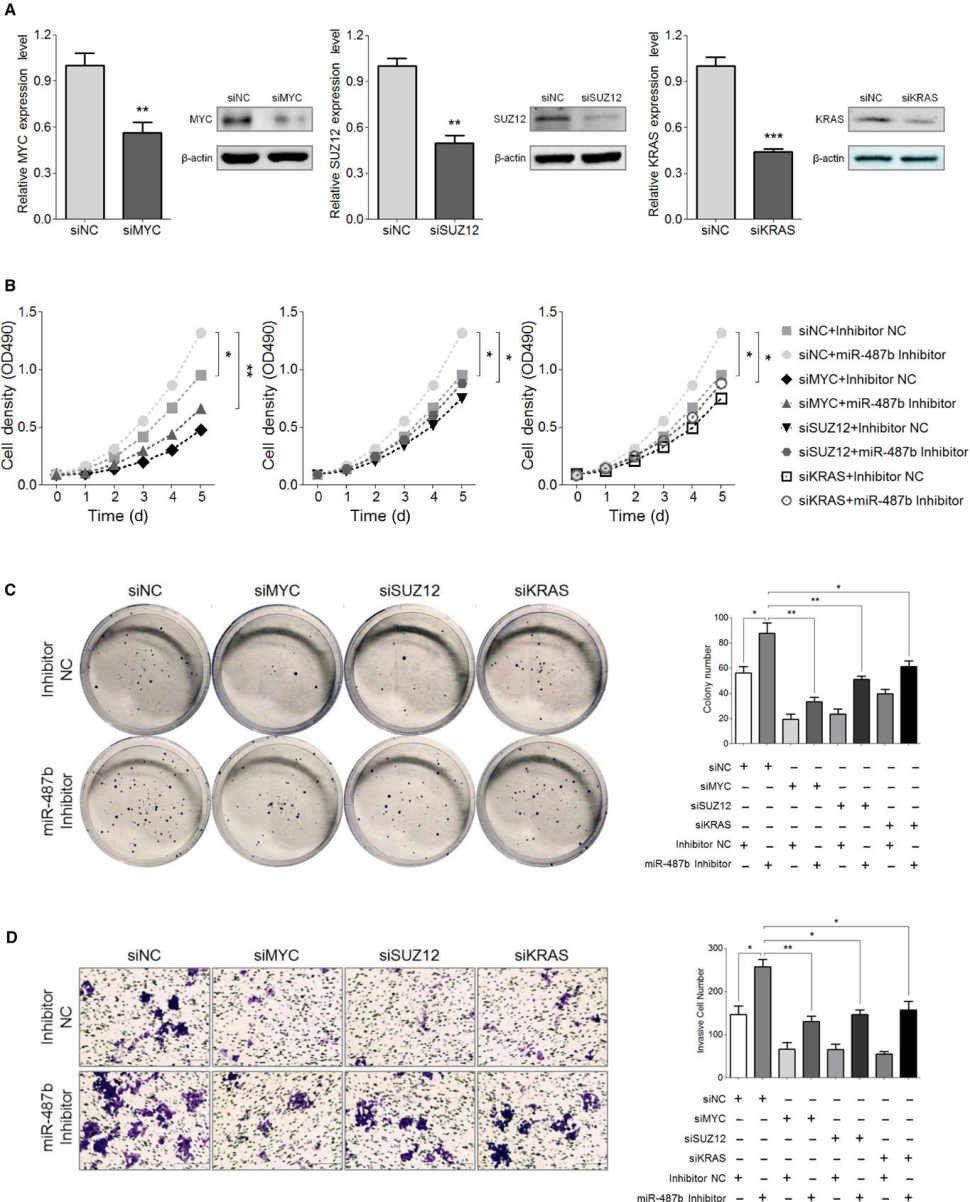
Inhibition of MYC/SUZ12/KRAS suppresses the miR‐487b inhibitor‐mediated colorectal cancer (CRC) progression. A, Expression levels of MYC, SUZ12, and KRAS in specific siRNA‐treated HCT116 cells compared with those in the NC groups at the mRNA and protein levels. B and C, Proliferative capacity of HCT116 miR‐487b inhibitor/NC cells with cotransfection of the MYC‐, SUZ12‐, or KRAS‐specific siRNAs, as indicated by MTT (B) and colony formation (C) assays. D, The migratory and invasive ability of HCT116 miR‐487b inhibitor/NC cells following transfection with specific siRNAs of MYC, SUZ12, or KRAS was evaluated via the Transwell assay. The *Scale bars* represent 100 μm. The data are presented as the means ± SD of at least three independent experiments. *P < 0.05, **P < 0.01, and ***P < 0.001

Motivated by our previous observation that miR‐487b could inhibit the expression of MYC, SUZ12, and KRAS at the mRNA and protein levels (Figure 4D,E), we continued to ask whether MYC, SUZ12, and KRAS were involved in the miR‐487b‐mediated suppressive effect. To study this assumption, we introduced the specific small interfering RNAs (siRNAs) and negative control (NC) of these three genes into our study. As shown in Figure 5A, MYC, SUZ12, and KRAS could effectively be decreased by specific siRNAs compared with the NC group at the mRNA and protein levels.

Based on the knockdown effects of siRNAs on MYC, SUZ12, and KRAS, we proceeded to explore whether the enhanced proliferative, metastatic, and invasive capabilities of miR‐487b inhibitor‐treated HCT116 cells could be restored compared with those in the NC group. Increasing proliferation caused by miR‐487b repression was partially abolished by a pool of small interfering RNAs of MYC, SUZ12, or KRAS in an MTT assay (Figure 5B). Additionally, the strengthened colony‐forming ability induced by miR‐487b inhibition was eliminated when MYC, SUZ12, or KRAS was simultaneously suppressed (Figure 5C). In addition, the silencing of MYC, SUZ12, or KRAS could neutralize the miR‐487b inhibitor‐mediated promotion of cell migration and invasion in the Transwell assay (Figure 5D). Together, these data suggest that miR‐487b suppresses CRC progression, at least in part by preventing the expression of MYC, SUZ12, or KRAS.

### 3.6 5‐Aza relieves the endogenous inhibition of miR‐487b in CRC cell lines

**Figure 6:**
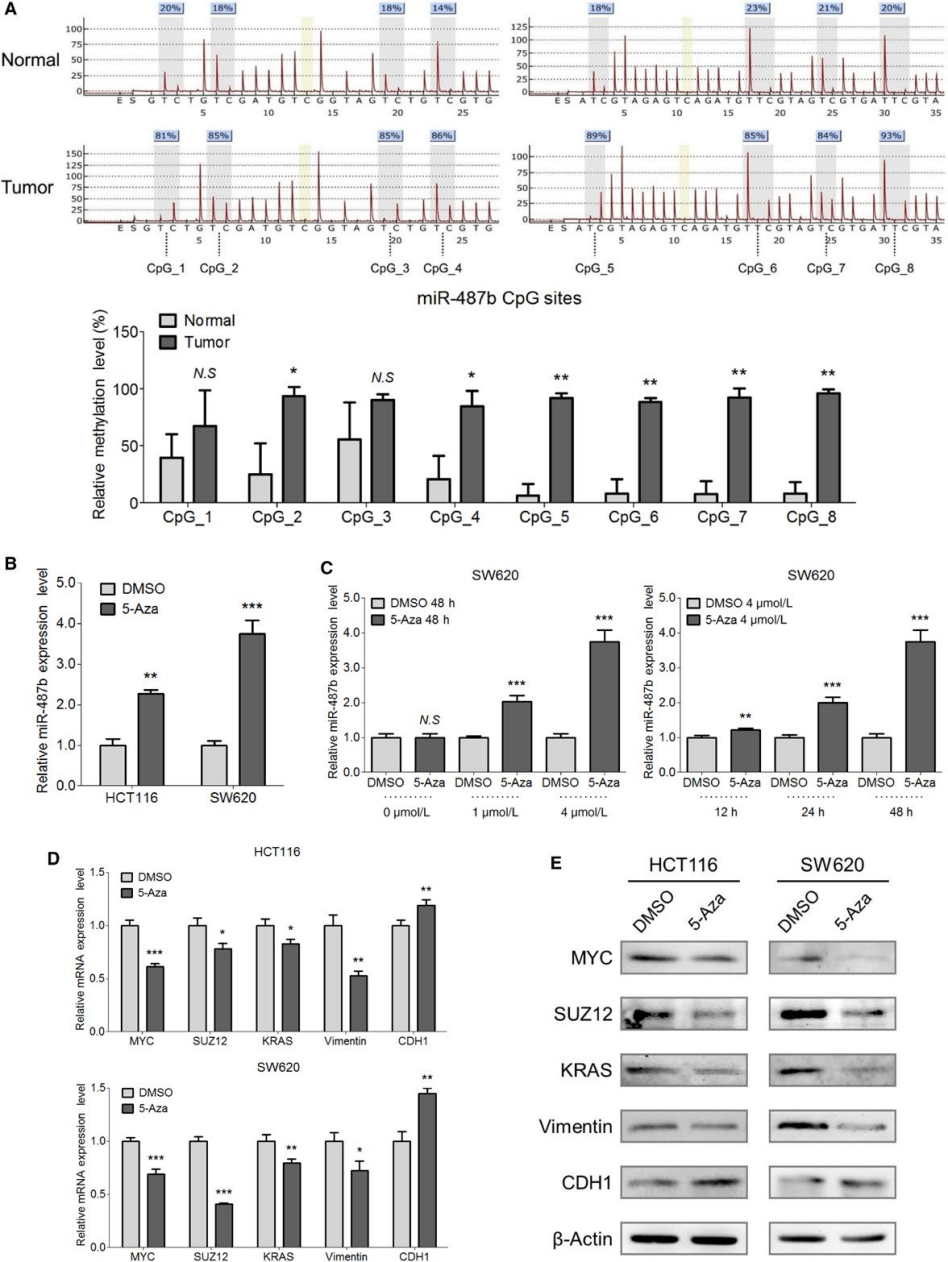
MiR‐487b is under the regulation of DNA methylation in colorectal cancer (CRC) cells. A, Methylation levels in the miR‐487b promoter region within the target sequences containing eight CpG sites in the three normal and CRC tissues were examined by pyrosequencing analysis, respectively. Representative results of specimens (*upper*) and statistical histogram (*lower*) are shown. B, qRT‐PCR analysis of miR‐487b expression in HCT116 and SW620 cells with 5‐Aza (4 μmol/L) treatment compared with that in the DMSO group. C, Different concentrations (0, 1, 4 μmol/L) and times (12, 24, 48 h) were applied to determine the effects of 5‐Aza on the miR‐487b expression in CRC cells. D and E, The mRNA and protein levels of miR‐487b biotargets (MYC, SUZ12, KRAS, Vimentin, and CDH1) were determined by qRT‐PCR (D) and Western blotting (E), respectively, in HCT116 and SW620 cells when treated with 5‐Aza/DMSO. The data are shown as the means ± SD of three independent experiments. NS, no significance, *P < 0.05, **P < 0.01, and ***P < 0.001

According to our former observation that miR‐487b was significantly restrained in both CRC cell lines (Figure 1A) and primary tumors (Figure 2C) compared with normal tissues, we hypothesized that a potential inhibiting factor existed during the transcription of miR‐487b in CRC tumorigenesis. Epigenetic modifications, especially DNA methylation, are implicated in multiple cancers and impair the transcriptional initiation of various tumor suppressive miRNAs.^25^ In this regard, we first detected the DNA methylation levels on the miR‐487b promoter region in normal and CRC tissues through pyrosequencing analysis. As shown in Figure 6A, compared with the normal tissues, the DNA methylation levels of the CpG_2, CpG_4, CpG_5, CpG_6, CpG_7, and CpG_8 sites were markedly increased in CRC tissues, indicating a DNA hypermethylated condition of the miR‐487b promoter, partially explaining the relatively low expression in the CRC patients.

Next, we used 5‐Aza, a DNA methyltransferase inhibitor, to investigate the mechanism of miR‐487b upstream regulation. We treated HCT116 and SW620 cells with 5‐Aza (4 μmol/L) for 48 hours and measured the expression level of miR‐487b in each cell line. 5‐Aza notably relieved transcription inhibition and promoted miR‐487b expression in both HCT116 and SW620 cells (Figure 6B). In addition, when exposed to 5‐Aza for different time periods (12, 24, and 48 hours) or concentrations (0, 1, and 4 μmol/L), the level of miR‐487b in SW620 cells gradually increased in a time‐ and dose‐dependent manner (Figure 6C). By contrast, when treated with 5‐Aza, the mRNAs of MYC, SUZ12, KRAS, and Vimentin were all dramatically reduced, whereas CDH1 mRNA was increased in HCT116 and SW620 cells (Figure 6D). Western blot analysis also revealed analogous protein changes in MYC, SUZ12, KRAS, Vimentin, and CDH1 after 5‐Aza induction (Figure 6E). Together, these data confirmed that miR‐487b is under the control of DNA methylation in CRC cells, suggesting removing DNA methylation might be a potential CRC therapeutic strategy in clinical applications.

## 4 DISCUSSION

MiRNAs function as critical modulators of human CRC tumorigenesis and malignant phenotypes. Through specific interactions with target mRNAs, miRNAs can play either onco‐miR or tumor suppressor roles. The miR‐154 gene family constitutes one of the largest miRNA clusters in the human genome, and several members of this family have been confirmed to play important roles in CRC. For example, by targeting TLR2, miR‐154 inhibits the ectopic proliferation and migration of CRC cells.^26^ Additionally, miR‐377, miR‐381, and miR‐409, another three members of the miR‐154 gene family, are involved in either invasion or drug sensitivity.^27^, ^28^ In this regard, as a member of the miR‐154 gene family, we have reasons to speculate miR‐487b is likely to play a role in cancer.

According to our observations, miR‐487b can prevent the overproliferation of CRC cells. Notably, the acceleration of apoptosis and deceleration of the cell cycle are two mechanisms for cell proliferation suppression. Consistent with Tsuyoshi Hata,^14^ we identified miR‐487b as a cell apoptosis promoter and KRAS inhibitor. However, we failed to identify its influence on the CRC cell cycle because there was no significant difference in the miR‐487b inhibitor‐ or mimic‐treated CRC cells compared with that in each NC group. Particularly, apart from KRAS, we also verified MYC and SUZ12 as the biotargets of miR‐487b in both CRC cells and patient specimens. Intriguingly, these three genes are all closely correlated with cell cycle progression in CRC. MYC triggers the stimulation of the CRC cell cycle by repressing p21.^30^ SUZ12 inhibits the levels of p16 and p21 via an H3K27me3‐mediated suppressive effect, ultimately launching CRC cell proliferation.^31^ KRAS‐associated genes exhibit functional enrichment in cell cycle and mitosis, and CDK4/6 inhibition is an effectively therapeutic response in KRAS‐dependent CRC.^32^ Because of this paradox, further investigations are still needed to clarify whether a negative feedback loop exists or if other cell cycle enhancement‐related genes are activated to block the miR‐487b‐mediated CRC cell cycle arrest. Currently, miR‐487b‐induced cell apoptosis elevation is the determining factor of CRC cell proliferation inhibition.

The epithelial‐mesenchymal transition (EMT) significantly contributes to the CRC development because normal intestinal epithelial cells can be vested with the malignant phenotypes during this process.^15^ In this study, we observed that miR‐487b could inhibit EMT morphology alterations of CRC cells, strengthen CDH1 and suppress Vimentin at the mRNA and protein levels both in vitro and in vivo. Nevertheless, this circumstance seems to be because of the lack of mediators due to the intrinsic function rather than because of regulatory genes such as CDH1 or Vimentin. In fact, MYC, SUZ12, and KRAS are all implicated in the regulation of EMT during CRC tumorigenesis. MYC facilitates the sLe (x/a) glycan in CRC cells to initiate the EMT process.^33^ SUZ12 is positively related to EMT characteristics in oxaliplatin‐resistant DLD1 cells.^34^ KRAS suppresses the miR‐200 family, which is the vital negative regulator of EMT in multiple human CRC subtypes.^35^ Furthermore, specific siRNAs of MYC, SUZ12, and KRAS can restore the miR‐487b inhibitor‐induced EMT phenotypes, further demonstrating that MYC, SUZ12, and KRAS are EMT regulatory mediators belonging to miR‐487b in CRC cells. Recently, Yi^13^ reported that miR‐487b participated in the interaction between GRM3 and TGF‐β, providing evidence to support our data highlighting the correlation between miR‐487b and the EMT process during CRC tumorigenesis.

Although small‐molecule compounds have been successively explored in recent decades and are widely used in different biological fields, only a small minority of these compounds is approved in disease therapy by the Food and Drug Administration (FDA). 5‐Aza, a representative candidate of these small compounds, is permitted to be coapplied with other drugs in different diseases, especially cancer.^36^ Additionally, as a DNA methyltransferase inhibitor (DNMTi), 5‐Aza was confirmed to be implicated in restraining the development and progression of CRC. 5‐Aza decreases the growth and increases the apoptosis of CACO2 cells by relieving the transcriptional limitation of RASSF1A.^37^ NALP1 can be restored by 5‐Aza to restrict CRC proliferation.^38^ Ultimately, 5‐Aza stimulates p53‐dependent tumor cell senescence and induces DNA double‐strand breaks.^39^ Furthermore, aberrant expression of miRNAs in CRC is frequently due to the inappropriate enrichment of epigenetic alterations, including DNA methylation and histone modification.^25^ Based on these clues, we attempted to build the connection between miR‐487b and this small‐molecule compound in CRC. First, we found that the miR‐487b promoter region was in a DNA hypermethylation condition in CRC tissues compared with normal ones. Next, the miR‐487b level was markedly upregulated in both HCT116 and SW620 cells in the presence of 5‐Aza. 5‐Aza was then proven to promote miR‐487b expression in a time‐ and concentration‐dependent manner. Finally, we compared the expression levels of MYC, SUZ12, KRAS, Vimentin, and CDH1 in HCT116 and SW620 cells under 5‐Aza treatment with each DMSO group and observed that 5‐Aza markedly suppressed the downstream genes of miR‐487b at both mRNA and protein levels. Notably, MYC, SUZ12, and KRAS can also be suppressed by 5‐Aza in other manners, especially with the involvement of miRNAs in different tumor microenvironments. For instance, 5‐Aza contributes to the upregulation of miR‐212 in gastric cancer,^40^ leading to the inhibition of MYC expression. 5‐Aza can also markedly reverse the aberrant level of miR‐200b,^41^ which may inhibit SUZ12 in cholangiocarcinoma.^42^ By removing the hypermethylation on the promoter regions of miR‐134,^43^ miR‐181c,^44^ and miR‐193b,^45^ 5‐Aza can restrain KRAS expression in the glioma, gastric carcinogenesis, and esophageal adenocarcinoma, respectively. However, our results did show that the inhibitory effect of 5‐Aza on MYC, SUZ12, and KRAS is at least partially achieved by miR‐487b in colon cancer cells. Therefore, our data strongly suggest that miR‐487b is under the control of DNA methylation, further providing an effective method to treat CRC by removing DNA methylation on the miR‐487b regulatory domains.

Tsuyoshi Hata^14^ revealed that the expression level of miR‐487b in normal tissues is notably lower than that in paired primary CRC tissues; however, our data showed an opposite result. We further clarified that miR‐487b was significantly downregulated in metastatic tissues compared with matched tumor tissues. Likewise, lower levels of miR‐487b can also be found in primary, high‐risk, or high‐stage tumor tissues than in normal specimens within the pulmonary carcinogenesis^11^ and neuroblastoma.^46^ Considering the above evidence, it is reasonable to further determine the applicable potential of miR‐487b as a diagnostic biomarker because miR‐487b acts as an antitumor and antimetastasis regulator of CRC in our study. Hence, we evaluated the clinical diagnosis value of miR‐487b through ROC curve analysis. MiR‐487b is useful to distinguish primary tumor tissues (T) from normal tissues (N) using an AUC (N/T) of 0.691 (*P* = 0.0029) and to differentiate metastatic tissues (M) from primary tumor tissues (T) with an AUC (T/M) of 0.689 (*P* = 0.0032) (Figure 2D). These findings are important for clinical applications because miR‐487b can be regarded as a biomarker for early CRC diagnosis and a criterion to judge metastasis.

**Figure 7:**
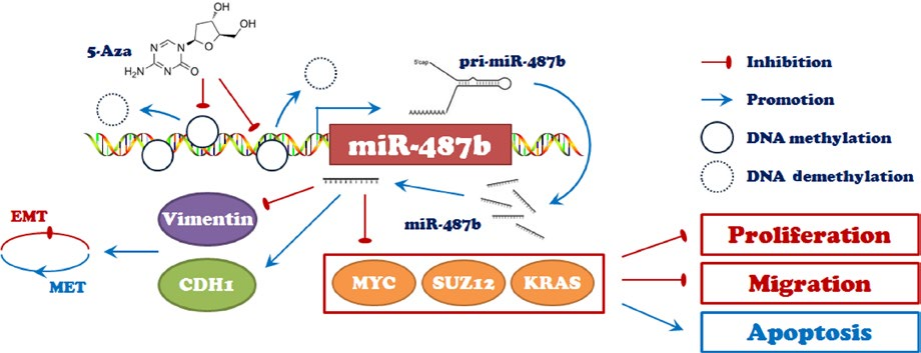
A schematic diagram is used to illuminate regulation in our study. MiR‐487b was forced to be inhibited due to high enrichment of DNA methylation on its promoter region, which can be partially reversed in the presence of 5‐Aza in colorectal cancer (CRC) cells. In addition, miR‐487b can suppress epithelial‐mesenchymal transition (EMT) through increasing CDH1 expression and decreasing Vimentin expression in vitro and in vivo. Additionally, miR‐487b can also repress MYC, SUZ12, and KRAS to promote apoptosis and restrain the proliferation and metastasis of CRC cells, thus indicating an intrinsic tumor suppressor role of miR‐487b in CRC

In summary, we confirmed that miR‐487b is a CRC suppressor that prevents the EMT process and targets MYC, SUZ12, and KRAS to inhibit the proliferation, migration and invasion of CRC cells. Meanwhile, miR‐487b is negatively regulated by DNA methylation (Figure 7). Hence, we identified miR‐487b as a diagnosis‐related biomarker and a novel target for CRC therapy.

## CONFLICT OF INTEREST

The authors declare that they have no conflict of interest in this manuscript.
