# Supplementary material for: DNA methylation‐regulated and tumor‐suppressive roles of miR‐487b in colorectal cancer via targeting MYC, SUZ12, and KRAS
Source: Cancer Med. 2019 Feb 21;8(4):1694–709. doi: 10.1002/cam4.2032 (PMC6488202; doi:10.1002/cam4.2032)
Supplement: Supplementary file 1 [file CAM4-8-1694-s001.docx]

**Supporting Information: Tables**

**Table S1**

Information on paired 41 CRC patients collected for clinical study

| **Patient Number** | **ID Number** | **Gender** | **Age** | **AJCC Pathologic TNM** | **Collected Tissue Specimens^1^** |
| --- | --- | --- | --- | --- | --- |
| 1 | D201103233 | Female | 61 | T4aN2b | N, T, L |
| 2 | D201109272 | Male | 62 | T3N2b | N, T, L |
| 3 | D201104503 | Male | 59 | T3N2b | N, T, L |
| 4 | D201105202 | Female | 43 | T3N1b | N, T, L |
| 5 | D201002192 | Female | 75 | T3N2Mx | N, T, L |
| 6 | D201002697 | Female | 44 | T3N2Mx | N, T, L |
| 7 | D201002527 | Female | 69 | T3N1Mx | N, T, L |
| 8 | D201311643 | Male | 66 | T3N2a | N, T, L |
| 9 | D201308052 | Female | 78 | T3N2a | N, T, L |
| 10 | D201302608 | Female | 65 | T4aN2aM1a | N, T, H |
| 11 | D201308960 | Male | 50 | T4aN1bM1 | N, T, H |
| 12 | D201205252  D201201687 | Male | 69 | T3N0Mx | N, T, H |
| 13 | D201503400 | Male | 38 | T4bN1a | N, T, L |
| 14 | D201502630 | Male | 71 | T4bN1a | N, T, L |
| 15 | D201501406 | Female | 27 | T4aN1bM1a | N, T, L |
| 16 | D201412114 | Male | 66 | T3N1a | N, T, L |
| 17 | D2014118161 | Male | 58 | T3N1a | N, T, L |
| 18 | D201411260 | Male | 75 | T4aN2b | N, T, L |
| 19 | D201411204 | Female | 50 | T3N1b | N, T, L |
| 20 | D201410386 | Male | 53 | T4aN2a | N, T, L |
| 21 | D201410268 | Male | 25 | T4aN1a | N, T, L |
| 22 | D201408938 | Male | 52 | T3N1b | N, T, L |
| 23 | D201406353 | Male | 23 | T4aN2b | N, T, L |
| 24 | D201405498 | Female | 54 | T3N1a | N, T, L |
| 25 | D201402883 | Male | 40 | T3N2a | N, T, L |
| 26 | D201401103 | Female | 53 | T3N2b | N, T, L |
| 27 | D201308257 | Female | 39 | T4bN1bM1 | N, T, L |
| 28 | D201300734 | Female | 71 | T3N1a | N, T, L |
| 29 | D201300516 | Male | 55 | T3N2b | N, T, L |
| 30 | D201204380 | Male | 74 | T4aN1a | N, T, L |
| 31 | D201201553 | Male | 50 | T4bN1b | N, T, L |
| 32 | D201103003 | Female | 59 | T3N1cM1 | N, T, H |
| 33 | D201002869 | Female | 76 | T4N1Mx | N, T, L |
| 34 | D201000430 | Male | 48 | T4N2M1 | N, T, H |
| 35 | D201308206  D201506178 | Female | 56 | T4aN0 | N, T, L |
| 36 | D201302048  D201501948 | Female | 61  63 | T3N1c | N, T, H |
| 37 | D201000011  D201410598 | Male | 57  61 | T3N0Mx | N, T, H |
| 38 | 200929911 | Female | 62 | T3N2Mx | N, T, L |
| 39 | 201002018 | Female | 53 | T4N1Mx | N, T, L |
| 40 | 200822027 | Female | 47 | T3N2b | N, T, L |
| 41 | 201210167  201205979 | Male | 62 | T3N0 | N, T, H |

^1^N: adjacent normal tissue, T: primary tumor tissue, L: metastatic lymph node tumor tissue, H: metastatic hepatic tumor tissue.

**Table S2**

Information on primer sequences used for qRT-PCR analysis

| **Gene** | **Forward (5’ - 3’)** | **Reverse (5’ - 3’)** |
| --- | --- | --- |
| miR-487b | AATCGTACAGGGTCATCCACTT | RR716, SYBR^®^ PrimeScript™ miRNA RT-PCR Kit, TaKaRa |
| CDH1 | TGCCCAGAAAATGAAAAAGG | GTGTATGTGGCAATGCGTTC |
| Vimentin | GAGAACTTTGCCGTTGAAGC | GCTTCCTGTAGGTGGCAATC |
| FN1 | CTGGCCGAAAATACATTGTAAA | CCACAGTCGGGTCAGGAG |
| ETS1 | ACAGGGTAAGTGAAGGTTAATTCCA | AGAAAGATGACTACCTTGCTTGACT |
| SNAI1 | ACAAGCACCAAGAGTCCG | ATGGCAGTGAGAAGGATGTG |
| SNAI2 | TCAGCTCAGGAGCATACAGCC | GGAGGAGGTGTCAGATGGAGG |
| ZEB1 | GACAGTGTTACCAGGGAGGAGCA | TTCAGGTGCCTCAGGAAAAATGA |
| ZEB2 | GGTATTGCCAACCCTCTG | CTCCCTTATTTCATCTTCCTCT |
| MYC | CCTCCACTCGGAAGGACTATC | TTCGCCTCTTGACATTCTCC |
| SUZ12 | CACGAGCTTTTCCTCCAGGCCT | AAACGTAAGCTGCAAATGAGCTGAC |
| KRAS | CGGTCATCCAGTGTTGTCAT | AATGCTCTTGATTTGTCAGCAG |
| U6 | CTCGCTTCGGCAGCACA | AACGCTTCACGAATTTGCGT |
| β-actin | TGGCATCCACGAAACTACC | GTGTTGGCGTACAGGTCTT |

**Table S3**

Information on the oligonucleotides sequences in this study

| **Oligonucleotides** | **Sense (5’ - 3’)** | **Antisense (5’ - 3’)** |
| --- | --- | --- |
| mimic NC/siNC | UUCUCCGAACGUGUCACGUTT | ACGUGACACGUUCGGAGAATT |
| miR-487b mimic | AAUCGUACAGGGUCAUCCACUU | GUGGAUGACCCUGUACGAUUUU |
| inhibitor NC | CAGUACUUUUGUGUAGUACAA |  |
| miR-487b inhibitor | AAGUGGAUGACCCUGUACGAUU |  |
| siMYC | CGAGCUAAAACGGAGCUUUTT | AAAGCUCCGUUUUAGCUCGTT |
| siSUZ12 | GCAUCUUAAACUCUGCCAUTT | AUGGCAGAGUUUAAGAUGCTT |
| siKRAS | GGGCUUUCUUUGUGUAUUUTT | AAAUACACAAAGAAAGCCCTT |

**Table S4**

Information on the antibodies used in this study

| **Antigen** | **Species** | **Applications and Dilutions** | **Source** |
| --- | --- | --- | --- |
| CDH1 | Rabbit | WB (1:500) | Santa Cruz Biotechnology #sc-7870 |
| Vimentin | Mouse | WB (1:500) | Santa Cruz Biotechnology #sc-66002 |
| MYC | Rabbit | WB (1:1000) | Cell Signaling Technology #9402 |
| SUZ12 | Rabbit | WB (1:1000) | Cell Signaling Technology #3737 |
| KRAS | Mouse | WB (1:500) | Santa Cruz Biotechnology #sc-30 |
| β-actin | Mouse | WB (1:2000) | Sigma-Aldrich #A1978 |
| Anti-rabbit IgG | Goat | WB (1:4000) | Cell Signaling Technology #7074 |
| Anti-mouse IgG | Goat | WB (1:4000) | Genshare #PB002H |

**Table S5**

Information on the reagents used in this study

| **Reagent** | **Company** | **Catalogue No.** |
| --- | --- | --- |
| 5-Aza-2’-Deoxycytidine (5-Aza) | Sigma-Aldrich | A3656 |
| Lipofectamine 2000 Transfection Reagent | Invitrogen | 11668019 |
| Propidium Iodide | Biolegend | 421301 |
| FITC Annexin V | Biolegend | 640906 |
